# Hantavirus Pulmonary Syndrome, French Guiana

**DOI:** 10.3201/eid1604.090831

**Published:** 2010-04

**Authors:** Séverine Matheus, Félix Djossou, David Moua, Anne Marie Bourbigot, Didier Hommel, Vincent Lacoste, Philippe Dussart, Anne Lavergne

**Affiliations:** Institut Pasteur de la Guyane, Cayenne, French Guiana (S. Matheus, D. Moua, V. Lacoste, P. Dussart, A. Lavergne); Centre Hospitalier Andrée Rosemon, Cayenne (F. Djossou, A.M. Bourbigot, D. Hommel)

**Keywords:** Hantavirus, pulmonary infection, zoonoses, rodent-borne, human, virus, French Guiana, letter

**To the Editor:** Hantaviruses are rodent-borne negative-sense RNA viruses belonging to the *Bunyaviridae* family, genus *Hantavirus*. Since the first report of a hantavirus in 1993 in the United States (*1*), different viruses belonging to this genus have been reported in the Americas (*2**–**5*). These New World viruses are responsible for a disease called hantavirus pulmonary syndrome (HPS), a respiratory illness caused by the inhalation of dust contaminated by rodent feces or urine containing the virus (*6**–**8*).

Until recently, no information was available concerning the presence of hantaviruses in French Guiana, a French overseas department (administrative unit) in South America. Nevertheless, the description of atypical pneumonia cases not related to any known etiologic agent and the identification of hantavirus reservoirs in neighboring countries led us to conduct a serologic study in a selected population of patients with compatible symptoms. The prevalence of immunoglobulin (Ig) G antibodies to hantavirus in this population was 1.42% (*9*). Subsequently, we systematically screened patients who had suggestive pathologies for hantavirus serology, which led us to the characterization of a divergent hantavirus.

On August 4, 2008, a 38-year-old man sought medical attention at the emergency department of Cayenne Hospital. He had had persistent symptoms of fever (>38.5°C), myalgia, diarrhea with melena, cough for 8 days, recurrent vomiting for 4 days, and dyspnea for 2 days. At consultation, tachypnea (respiratory rate 28/min) and oxygen desaturation (SaO_2_ 83%) were observed. Chest radiograph showed bilateral diffuse interstitial infiltrates causing respiratory distress; mechanical ventilation was required. The patient was admitted to the intensive care unit for treatment of acute respiratory distress syndrome. Results of laboratory investigations performed when the patient was admitted showed thrombocytopenia (50,000 cells/mm^3^), leucocytosis (22,500 cells/mm^3^) associated with a high neutrophil count (20,300 cells/mm^3^), moderate hepatonephritis (alanine aminotransferase 17 IU/L, aspartate aminotransferase 31 IU/L, gamma-glutanyl transferase 44 IU/L; alkaline phosphatase 44 IU/L; creatinine 192 µmol/L and urea 9.3 mmol/L); and an elevated C-reactive protein concentration (>192 mg/L). Laboratory tests for infectious agents ruled out malaria, dengue, leptospirosis, Chagas disease, Q fever, cytomegalovirus, and HIV, and blood cultures were negative for bacterial growth. The patient remained under respiratory assistance for 25 days in the intensive care unit and was discharged from hospital 47 days after admission with a complete clinical recovery.

With no etiologic agent identified, 2 factors led to the suspicion of hantavirus infection: clinical symptoms compatible with HPS and the patient’s exposure to potential reservoirs. Indeed, a month before the onset of symptoms, he had moved to a rural municipality located near agricultural lands and forest.

Retrospective serologic investigations were performed with the 3 available serum samples obtained during the hospitalization. These samples were tested by IgM capture with inactivated Sin Nombre virus antigens and by indirect ELISA with recombinant antigens to detect IgG antibodies to Sin Nombre virus (*10*). IgM to Sin Nombre virus were present in the samples collected 8 and 9 days, respectively, after onset of the disease, confirming hantavirus infection. Furthermore, IgG to Sin Nombre virus were only detected in the convalescent-phase serum samples obtained on day 41 of the disease. These serologic results suggested a recent infection with hantavirus.

Molecular investigations were performed to characterize and identify the virus. Viral RNA was extracted from the 2 acute serum samples. Reverse transcription-PCR was performed with consensus primers targeting the S segment of the hantavirus genome as described in Johnson et al. (*4*). Amplification products of the expected size (434 bp of the nucleoprotein N-encoding region) were obtained from both samples. Cloning and sequencing of these products allowed obtaining a consensus sequence, which was deposited with GenBank (GQ179973). Database searches using BLAST (www.ncbi.nlm.nih.gov/blast) demonstrated that this sequence, although novel, is most similar to Rio Mamore hantavirus strain OM-556 (GenBank accession no. U52136), showing 83% nucleotide identity (393 bp analyzed, excluding the primers). In addition, comparison with representative hantavirus sequences from New World isolates showed that the amplified fragment exhibited from 73.5% to 81.9% nucleotide sequence identity and from 90.1% to 96.9% amino acid sequence identity (Table). This level of sequence divergence, as well as the geographic specificity of this hantavirus in French Guiana led us to provisionally name it Maripa virus.

**Table:** Comparison of nucleotide and deduced amino acid sequences between Maripa virus and representative New World hantaviruses*

Maripa virus	% Nucleotide sequence identity	% Amino acid sequence identity
RIOM_B	83.0	96.9
RIOM_P	81.9	96.2
ANAJ	81.2	96.2
LAN	81.2	96.2
RIME	77.9	94.7
PARN	78.4	93.9
ARAQ	80.2	93.1
AND	78.6	93.1
ORN	78.4	93.1
MCL	76.8	93.1
CHO	76.1	93.1
PRG	76.6	93.1
LEC	80.2	92.4
BMJ	79.4	92.4
CAD	77.1	92.4
ARAC	77.6	91.6
C_Plata	77.6	90.8
SN	73.5	90.1

*RIOM_B, Rio Mamoré virus strain OM-556 (U52136); RIOM_P, Rio Mamoré virus strain HTN-007 (AF133254); ANAJ, Anajatuba virus isolate Of58 (DQ451829); LAN, Laguna Negra virus strain 510B (AF005727); RIME, Rio Mearim virus isolate Hs85 (DQ451828); PARN, Paranoa virus (EF576661); ARAQ, Araraquara virus (AF307325); AND, Andes virus strain AH-1 (AF324902); ORN, Oran virus strain 22996, (AF482715); MCL, Maciel virus strain 13796 (AF482716); CHO, Choclo virus (DQ 285046); PRGm Pergamino virus strain 14403 (AF482717); LEC, Lechiguanas virus strain 22819 (AF482714); BMJ, Bermejo virus strain Oc22531 (AF482713); CAD, Cano Delgadito virus isolate VHV-574 (AF000140); ARAC, Araucaria virus strain HPR/04-102 (AY740633); CAS, Castelo dos Sonhos virus (AF307324); C_Plata, Central Plata virus strain 714LC (EU564715); SN, Sin Nombre virus strain NM H10 (L25784).

Results of a serologic survey to identify cases of respiratory disease with no evident etiology led us to identify an HPS case-patient in French Guiana who had been infected with a new divergent hantavirus strain. Human hantavirus epidemics are associated with fluctuations of rodent populations caused by climatic, ecologic and environmental changes or with changes in human activities associated with nature or agriculture. Therefore, in this region where 90% of the land is tropical rain forest but in which there is increasing economic development, continuous surveillance for the virus in the human population would be beneficial. Surveys of potential reservoirs may help reduce the risk of viral emergence.
